# Performance of recombinant chimeric proteins in the serological diagnosis of *Trypanosoma cruzi* infection in dogs

**DOI:** 10.1371/journal.pntd.0007545

**Published:** 2019-06-26

**Authors:** Leonardo M. Leony, Natália E. M. Freitas, Rodrigo P. Del-Rei, Claudia M. Carneiro, Alexandre B. Reis, Ana Maria Jansen, Samanta C. C. Xavier, Yara M. Gomes, Edmilson D. Silva, Mitermayer G. Reis, Deborah B. M. Fraga, Paola A. F. Celedon, Nilson I. T. Zanchin, Filipe Dantas-Torres, Fred L. N. Santos

**Affiliations:** 1 Gonçalo Moniz Institute, Oswaldo Cruz Foundation, Salvador, Bahia, Brazil; 2 Faculty of Technology and Sciences of Bahia, Salvador, Bahia, Brazil; 3 Immunopathology Laboratory, Nucleus of Research in Biological Sciences, Federal University of Ouro Preto, Ouro Preto, Minas Gerais, Brazil; 4 Oswaldo Cruz Institute, Oswaldo Cruz Foundation, Rio de Janeiro, Rio de Janeiro, Brazil; 5 Aggeu Magalhães Institute, Oswaldo Cruz Foundation, Recife, Pernambuco, Brazil; 6 Immunobiological Technology Institute, Rio de Janeiro, Rio de Janeiro, Brazil; 7 Department of Pathology and Legal Medicine, Federal University of Bahia, Bahia, Brazil; 8 Department of Epidemiology of Microbial Diseases, School of Public Health, Yale University, New Haven, Connecticut, United States of America; 9 Molecular Biology Institute of Paraná, Curitiba, Paraná, Brazil; 10 Carlos Chagas Institute, Oswaldo Cruz Foundation, Curitiba, Paraná, Brazil; Tulane University, UNITED STATES

## Abstract

**Background:**

Dogs are considered sentinels in areas of *Trypanosoma cruzi* transmission risk to humans. ELISA is generally the method of choice for diagnosing *T*. *cruzi* exposure in dogs, but its performance substantially depends on the antigenic matrix employed. In previous studies, our group has developed four chimeric antigens (IBMP-8.1, 8.2, 8.3, and 8.4) and evaluated their potential for diagnosing *T*. *cruzi* exposure in humans. For human sera, these chimeric antigens presented superior diagnostic performances as compared to commercial tests available in Brazil, Spain, and Argentina. Therefore, in this study we have evaluated the potential of these antigenic proteins for detection of anti-*T*. *cruzi* IgG antibodies in dog sera.

**Methodology/Principal findings:**

The IBMP-ELISA assays were optimized by checkerboard titration. Subsequently, the diagnostic potential was validated through analysis of ROC curves and the performance of the tests was determined using double entry tables. Cross-reactivity was also evaluated for babesiosis, ehrlichiosis, dirofilariosis, anaplasmosis, and visceral leishmaniasis. Best performance was shown by IBMP-8.3 and IBMP-8.4, although all four antigens demonstrated a high diagnostic performance with 46 positive and 149 negative samples tested. IBMP-8.3 demonstrated 100% sensitivity, followed by IBMP-8.4 (96.7–100%), IBMP-8.2 (73.3–87.5%), and IBMP-8.1 (50–100%). The highest specificities were achieved with IBMP-8.2 (100%) and IBMP-8.4 (100%), followed by IBMP-8.3 (96.7–97.5%) and IBMP 8.1 (89.1–100%).

**Conclusions/Significance:**

The use of chimeric antigenic matrices in immunoassays for anti-*T*. *cruzi* IgG antibody detection in sera of infected dogs was shown to be a promising tool for veterinary diagnosis and epidemiological studies. The chimeric antigens used in this work allowed also to overcome the common hurdles related to serodiagnosis of *T*. *cruzi* infection, especially regarding variation of efficiency parameters according to different strains and cross-reactivity with other infectious diseases.

## Author summary

Despite dogs being considered *T*. *cruzi*’s most important domestic sentinel/reservoir and also suffering from the outcomes of the infection, there has never been a commercially available test to diagnose *T*. *cruzi* infection in dogs. As such, our group’s objective was to develop a state-of-the-art serological diagnostic test utilizing four chimeric antigens (IBMP-8.1, 8.2, 8.3, and 8.4) in an ELISA platform to accurately identify canine anti-*T*. *cruzi* IgG antibodies. The IBMP-ELISA assays were optimized, evaluated for cross-reactivity towards multiple canine parasite’s, including *Leishmania* spp., its diagnostic potential was validated and the test’s performance was determined using double entry tables. The IBMP-8.3 antigen demonstrated 100% sensitivity, followed by IBMP-8.4, whereas the highest specificities were achieved with IBMP-8.2 (100%) and IBMP-8.4 (100%). Therefore, we’ve concluded that the serodiagnosis through anti-*T*. *cruzi* IgG detection in dogs, utilizing chimeric antigenic matrices in immunoassays is a promising tool for veterinary diagnosis and epidemiological surveillance. Furthermore, the use of chimeric antigens efficiently addressed common hurdles related to *T*. *cruzi* serodiagnosis, especially regarding efficiency variation in response to different strains and cross-reactivity.

## Introduction

Chagas disease (CD) is a vector-borne, neglected parasitic illness caused by the hemoflagellate protozoan *Trypanosoma cruzi*. According to recent estimates, approximately 6 million people are affected by CD in 21 Latin American endemic countries with ~14,000 deaths per year being attributed to this disease [1]. Increased international migration of infected individuals has spread CD toward non-endemic settings, including North American, European, Asian, and Oceanian countries [2,3].

*Trypanosoma cruzi* transmission involves complex networks of interactions of wild and domestic mammalian hosts, and triatomine vectors. Dogs, cats, pigs, and goats are the main domestic mammalian species investigated for *T*. *cruzi* infection in endemic areas. Dogs and cats represent the first domestic *T*. *cruzi* hosts studied by Carlos Chagas: a cat in Lassance (Minas Gerais state, Brazil) was the first mammalian host in which he found trypomastigote forms of the parasite in the blood, whereas dogs were among the first experimental models used in his research. Since then, several studies have shown that dogs and cats can be competent *T*. *cruzi* reservoirs, but as described for the other mammals, their importance in the transmission cycle varies according to the geographic regions and local characteristics. Particularly, domestic dogs have been commonly implicated as blood meal sources for triatomine vectors [4,5]. In the Argentinean Gran Chaco, both dogs and cats are epidemiologically important and described as highly infective to triatomine vectors [4,5]. Active transmission, which includes symptomatic dogs, was also observed in the southern United States [6,7] and throughout the Americas [8]. A different scenario is seen in Brazil. Despite being exposed to parasite (as evidenced by the high seropositive rates by IFAT and ELISA), *T*. *cruzi* isolation from dogs, whether by hemoculture or xenodiagnoses, is rarely documented [6–8]. However, dogs may act as efficient sentinel animals. Xavier et al. [7] observed positive association between serologically positive dogs and: (1) lower diversity of small mammal fauna and (2) high rates of small mammal fauna with high infective competence as expressed by positive hemocultures.

The presence of *T*. *cruzi*-infected dogs in households is associated with higher risk of human infection; as such, they can be used as sentinels [9]. Furthermore, dogs may eventually develop American trypanosomiasis (AT) by *T*. *cruzi*, presenting morphofunctional cardiac lesions or sudden death similar to those seen in humans [10,11]. Hence, AT may represent a health risk for dogs [12]. The pivotal importance is that seropositive dogs reflect exposure to *T*. *cruzi* and indicate the presence of this parasite in areas where these animals roam. The knowledge of *T*. *cruzi* infection in these hosts may direct epidemiological measures to risk settings even before the occurrence of human cases.

Despite the importance of dogs in the epidemiological scenario of *T*. *cruzi* transmission, there are no commercial serological tests to diagnose canine *T*. *cruzi*-infection. Several studies employed conventional in-house ELISA to diagnose *T*. *cruzi* exposure in dogs, which uses either fractionated *T*. *cruzi* lysates or whole-cell epimastigote homogenates as antigenic matrices [10,13–15]. This complex antigenic mixture of the variable component is highly sensitive and some drawbacks have been already described, such as difficult standardization, low specificity, and cross-reactivity with *Leishmania* spp., and other trypanosomatids [16,17]. Therefore, accurate serological diagnostic tests are needed to fill this gap.

Multi-epitope recombinant proteins, composed of several antigenically distinct amino acid sequences, have been proposed to improve the diagnostic performance of human chronic CD diagnosis [18–23]. Four multi-epitope proteins, namely IBMP-8.1, IBMP-8.2, IBMP-8.3, and IBMP-8.4, have been expressed by our team and their diagnostic potential has previously been evaluated on several technical platforms to diagnose human CD [18–20]. These antigens presented high levels of sensitivity, specificity, and accuracy for samples from both endemic and non-endemic areas for several geographic regions [19,24,25]. Cross-reactivity has already been evaluated and only a small number of samples were classified as reagent for various infectious diseases of clinical interest, including leishmaniasis [19,26]. The results obtained for human samples are very promising. Therefore, in this study, we aimed to assess the efficiency of these multi-epitope proteins as an antigenic matrix in serological assays for anti-*T*. *cruzi* IgG antibody detection in dogs with chronic *T*. *cruzi* infection.

## Materials and methods

### Ethical statements

This investigation was approved by the Animal Ethical Committee from Gonçalo Moniz Institute, Oswaldo Cruz Foundation, Salvador, Bahia, with the number 002/2017. The samples were provided by the biorepositories of the Trypanosomatid Biology Laboratory (Fiocruz, Rio de Janeiro), Immunopathology Laboratory of the Nucleus of Research in Biological Sciences (NUPEB—Federal University of Ouro Preto–UFOP), Host-Parasite Interaction and Epidemiology Laboratory (Fiocruz-BA), and Immunoparasitology Laboratory (Fiocruz-Pernambuco).

### Chimeric proteins synthesis

**Fig 1:**
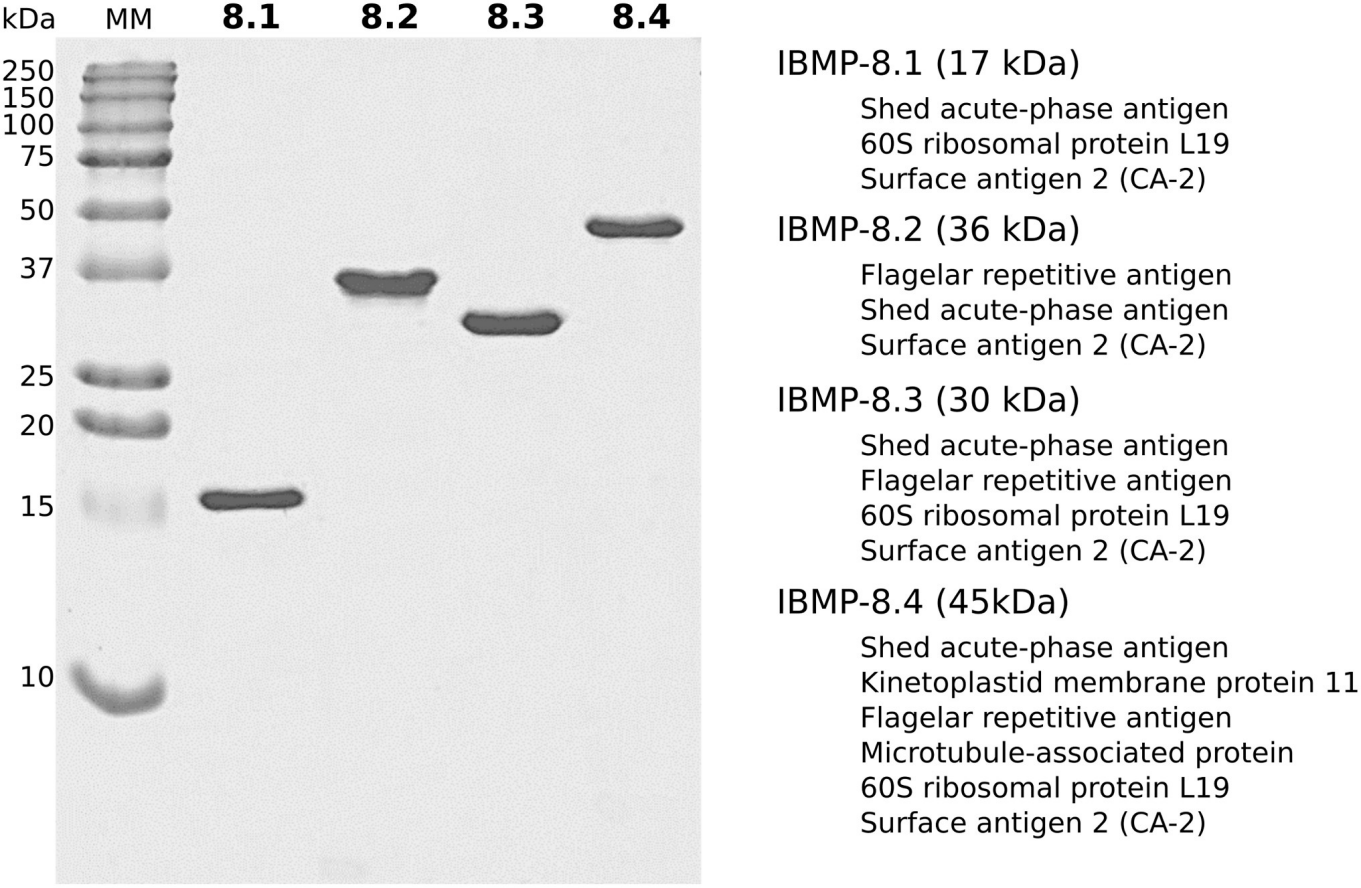
SDS-PAGE stained with Coomassie brilliant showing the chimeric purified antigens. The *T*. *cruzi* proteins whose antigenic regions were used to construct the chimeric antigens are described on the right. Lanes 8.1 to 8.4 indicate the IBMP multi-epitope antigens (1 μg of each antigen was loaded per lane). MM: molecular weight marker.

The multi-epitope antigens used in the indirect ELISA were obtained according to Santos *et al*. [18]. Briefly, the synthetic genes were subcloned into the pET28a vector and expressed in *Escherichia coli* BL21-Star (ThermoFisher Scientific). Expression was induced with 0.5 μM of IPTG (isopropyl β-D-1-thiogalactopyranoside) and the soluble proteins purified by both ion exchange and affinity chromatography. Finally, the purified muti-epitope antigens were quantified by Qubit fluorometric assay (ThermoFisher Scientific). Fig 1 illustrates the SDS-PAGE of the antigens after purification. Details about IBMP composition has been seen in previous studies of our group [18,19,23].

### Sample collections

**Fig 2:**
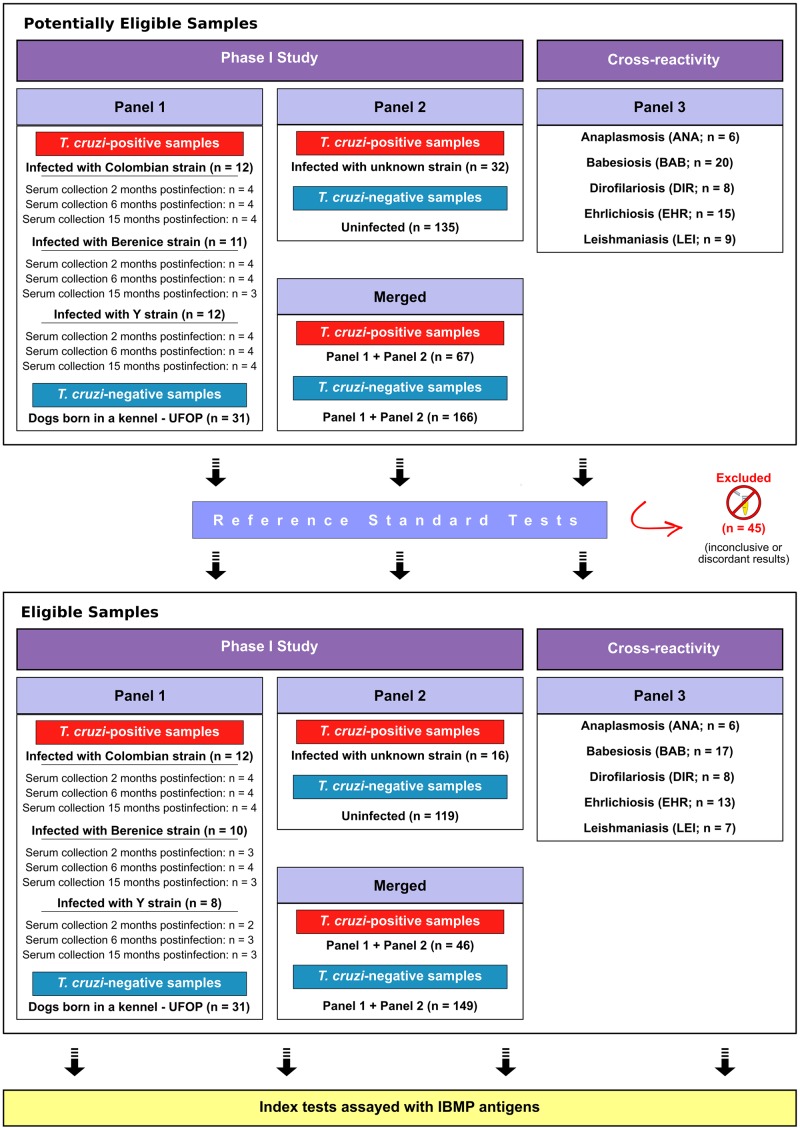
STARD flowchart to evaluate the diagnostic performance of IBMP chimeric antigens to detect anti-*Trypanosoma cruzi* antibodies in dog sera. Reference Standard Tests: in-house ELISA using fractionated lysates of *T*. *cruzi* at the epimastigote and modified Gold ELISA Chagas (Rem Indústria e Comércio, Brazil).

Three convenience canine serum panels from dogs of different origins were used in this investigation (Fig 2). The first panel (panel 1) was composed of either non-infected (n = 31) or experimentally infected mongrel dogs with the Colombian (n = 12), Y (n = 8) and Berenice (n = 10) strains of *T*. *cruzi*. These samples were obtained from animals born and maintained at UFOP’s Animal Science Center kennel, Ouro Preto, Minas Gerais, Brazil. Serum samples from experimentally infected dogs were collected 2–15 months after *T*. *cruzi* inoculation. The second panel (panel 2), composed of *T*. *cruzi*-negative (n = 119) and positive (n = 16) samples, was provided by the biorepository of the Trypanosomatid Biology Laboratory (Fiocruz-RJ) or the Host-Parasite Interaction and Epidemiology Laboratory (Fiocruz-BA). These samples were obtained from previous investigations and were collected in several *T*. *cruzi* endemic settings from Brazil [27–29]. Combined analysis of panel 1 and panel 2 was denominated as “Merged.” In addition to *T*. *cruzi*-positive and negative sera, 51 samples from animals with unrelated pathogens infection (panel 3) were combined into the sample set to assess cross-reactivity. The unrelated pathogens infection evaluated included anaplasmosis (n = 6), babesiosis (n = 17), dirofilariosis (n = 8), and ehrlichiosis (n = 13). These samples were from the biorepository of the Immunoparasitology Laboratory (Fiocruz-PE) and previously characterized by molecular and serological methods [27]. Additionally, sera from mongrel dogs experimentally infected with *Leishmania infantum* (n = 7) were provided by the Immunopathology Laboratory (UFOP) and assessed herein [30].

### Sera characterization

All samples were initially re-assayed for IgG antibodies against *T*. *cruzi* using two in-house ELISA. In the first test, 100 μl of fractionated lysates of *T*. *cruzi* at the epimastigote stage (2.4 μg/ml) in carbonate-bicarbonate buffer (50 mM, pH 9.6) was used to sensitize the microplates for 60 min at 37°C. Following washing steps with PBS 0.05% Tween 20 (PBS-T), the microplates were blocked with phosphate-buffered saline (PBS) supplemented with 2% milk lecithin for 30 min at 37°C. After washing, 100 μl of anti-dog IgG-HRP conjugated (Sigma, St. Louis, USA), loaded at 1:40.000 in PBS, were added and the microplates incubated for 30 min at 37°C. Following incubation and washing cycles, 100 μl of TMB substrate (tetramethyl-benzidine; Sigma, St. Louis, USA) were added, and the microplates were incubated in the dark at room temperature (RT) for 30 min. Then, the enzymatic reactions were stopped by adding 50 μl of 0.5 M H_2_SO_4_, and the optical density (OD) at 450 nm was read in a MultiskanFC microplate spectrophotometer (Thermo Scientific, Finland). The other test refers to a modified Gold ELISA Chagas kit (Rem Indústria e Comércio, São Paulo, Brazil). In this test, dilutions of anti-dog IgG-HRP conjugate tested were 1:20,000, 1:40,000, and 1:80,000 (Bio-Manguinhos, Rio de Janeiro, Brazil). Similarly, *T*. *cruzi*-positive and negative serum samples were assayed at dilutions 1:100, 1:200, 1:400, and 1:800. The best conditions to separate negative and positive samples (delta median—Δ) were conjugated antibody diluted at 1:40,000 and serum dilution at 1:800. The cut-off (CO) was established by using the mean optical absorbance of negative sera plus three standard deviations. If a sample’s optical density (OD) value fell within ± 10% of the CO value, it was considered as an indeterminate result (or in the grey zone). Samples with repeatedly discrepant results between both tests or inconclusive in one of them (or within the gray zone) were excluded. These two in-house ELISA were used as reference tests to determine the presence of IgG anti-*T*. *cruzi* antibodies in the investigated samples. Each sample was given an identifier code in the laboratory to ensure a blinded analysis.

### IBMP-ELISA optimization and procedure

The optimal dilutions of the antigen coating, as well as the dilutions of the antibody-enzyme conjugate (HRP) and serum concentrations, were determined by cross-titration. The selected conditions were established considering the largest difference in the average optical density (OD) value between positive and negative samples. The conditions were considered satisfactory when negative samples’ OD averaged below or around 0.25 and positive samples above or next to 1.00. The selected conditions for each chimeric antigen were established considering the highest difference between the median OD for positive and negative *T*. *cruzi* samples (delta median—Δ). Flat bottom, high-binding, transparent “Maxisorp” 96-well microplates (Nunc, Roskilde, Denmark) were coated with IBMP antigens (12.5 ng, 25 ng, and 50 ng) in carbonate-bicarbonate buffer (50 mM, pH 9.6). Following the blocking step with Well Champion reagent (Kem-En-Tec, Taastrup, Denmark), 100 μl of a serial dilution of each serum sample (1:100 and 1:200) diluted in phosphate-buffered saline (pH 7.4) was added to the selected well and the microplate incubated at 37 °C for 60 min. After washing with phosphate-buffered saline-0.05% Tween 20, 100 μL of HRP-conjugated goat anti-dog IgG (Fiocruz, Rio de Janeiro, Brazil), diluted at 1:20,000, 1:40,000, and 1:80,000 ratios in phosphate-buffered saline, were added to the wells and the microplate incubated at 37°C for 30 min. After another washing cycle, 100 μl of TMB substrate (tetramethyl-benzidine; Kem-En-Tec, Taastrup, Denmark) were added to each well and the microplates incubated in the dark at RT for 10 min. The reaction was interrupted by adding 50 μl of 0.3 M H_2_SO_4_ to each well. The OD was measured using a 450nm filter (SPECTRAmax 340PC, USA). CO values were established under ROC curve analysis. The results were normalized as a reactivity index (RI) that denotes the ratio between the OD of the samples and the CO. Samples that resulted in RI > 1.0 were considered positive. If a sample’s RI value was within ± 10% of 1.0, it was classified as inconclusive (or in the grey zone). Each sample was given an identifier code in the laboratory to ensure a blinded analysis.

### Statistical analysis

Data were analyzed using a scatter plot graphing software (GraphPad Prism version 7, San Diego, CA, USA). Continuous variables were presented as geometric mean ± standard deviation (SD). The Shapiro-Wilk test was used to test data normality. When the assumed homogeneity was confirmed, Student’s t-test was used. If not, Wilcoxon’s signed-rank test was employed. All analyses were two-tailed, and p values under 5% were considered significant (p < 0.05). Areas under the ROC curve (AUC) were calculated to assess the global accuracy for each IBMP antigen, which can be classified as outstanding (1.0), elevated (0.82–0.99), moderate (0.62–0.81), or low (0.51–0.61) [31]. IBMP-ELISA performance parameters were determined using a dichotomous approach and compared regarding sensitivity (Se), specificity (Sp), and accuracy (Ac). A 95% confidence interval (95% CI) was calculated to address precision of the proportion estimates. The agreement strength between the reference standard tests and IBMP-ELISA was established by Cohen’s kappa (κ) analysis, which was interpreted as follows: 1.0 ≤ κ ≥ 0.81 (almost perfect agreement), 0.80 ≤ κ ≥ 0.61 (substantial agreement), 0.60 ≤ κ ≥ 0.41 (moderate agreement), 0.40 ≤ κ ≥ 0.21 (fair agreement), 0.20 ≤ κ ≥ 0 (slight agreement), and k = 0 (poor agreement) [32]. A checklist (S1 Table) and flowchart (Fig 2) are provided according to the Standards for the Reporting of Diagnostic accuracy studies (STARD) guidelines.

## Results

### Chimeric antigen-ELISA optimization

The optimal dilutions of sera, antigens and antibody-enzyme conjugate were assessed by checkerboard titration. The best condition was chosen by considering the higher Δ median between *T*. *cruzi*-positive and negative samples. The pre-established criteria (OD < 0.25 for negative samples and OD > 1.00 for *T*. *cruzi*-positive samples) conditions were classified as satisfactory with the antibody-enzyme conjugate at a dilution of 1:20.000 for IBMP-8.3 and 1:40.000 for IBMP-8.1, IBMP-8.2, and IBMP-8.4. With respect to serum dilution, all tests presented higher Δ median when diluted at 1:100 compared to 1:200). Conversely, the best quantity of antigen to sensitize each well varied from 25 ng for IBMP-8.1, IBMP-8.2, and IBMP-8.4 to 50 ng for the IBMP-8.3.

### Chimeric antigen-ELISA performance

**Fig 3:**
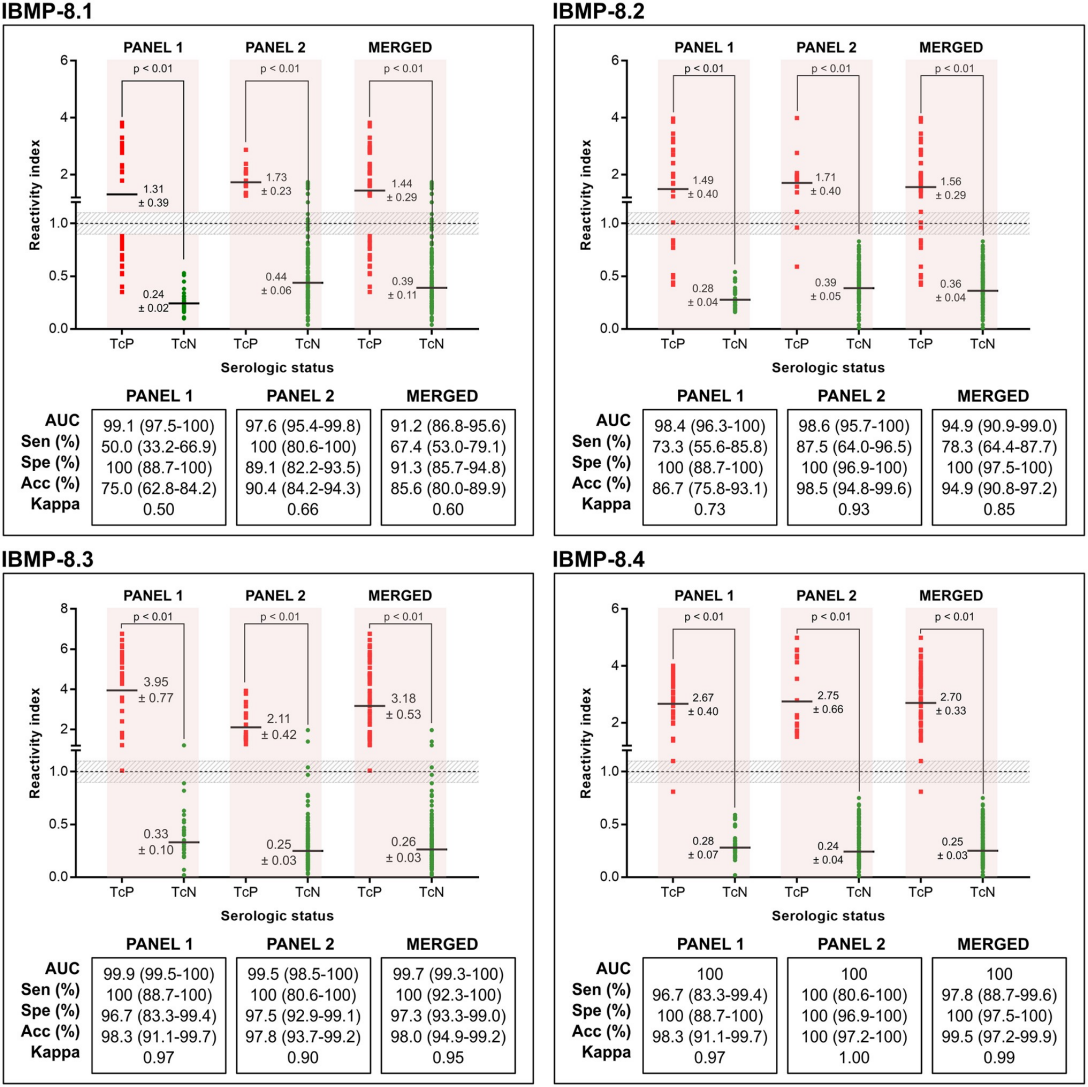
Reactivity index and diagnostic performance parameters obtained with serum samples from *T*. *cruzi*-infected and non-infected dogs. **Panel 1 (dogs experimentally infected with Y, Berenice, and Colombian *T*. *cruzi* strains); Panel 2 (dogs naturally infected with unknown *T*. *cruzi* strains).** The cut-off is set at the reactivity index value = 1.0 and the shadowed area represents the grey zone (RI = 1.0 ± 0.10). Horizontal lines and numbers for each result group represent the geometric means (± 95% CI). Acc (accuracy); AUC (area under curve); Sen (sensitivity); Spe (specificity); TcP (*T*. *cruzi*-positive samples); TcN (*T*. *cruzi*-negative samples).

The phase I study was performed using two distinct serological panels. Panel 1 was composed of sera from dogs experimentally infected with three known strains of *T*. *cruzi*, whereas panel 2 was formed by sera from dogs naturally infected with unknown strains of *T*. *cruzi*. The merged analysis was also performed considering the samples from both panels 1 and 2 (Fig 3; individual data points are available in the S2 Table). Based on AUC values, all IBMP chimeric antigens were classified with either high or outstanding diagnostic potential, regardless of serum panel assayed. However, AUC values for IBMP-8.3 and IBMP-8.4 were statistically higher than those for IBMP-8.1 and IBMP-8.2. For *T*. *cruzi*-positive samples, IBMP-8.3 produced the highest RI value for merged analysis. No significative difference was observed between IBMP-8.3 and IBMP-8.4, considering overlap of 95% CI values. The lowest RI value was seen for IBMP-8.1. However, no differences were shown between IBMP-8.1 and IBMP-8.3 or IBMP-8.2 considering only panel 2. With respect to *T*. *cruzi*-negative samples, RI values were below 0.45 for all four chimeric antigens in all investigated panels.

IBMP-8.3 presented a sensitivity of 100% for all panels, followed by IBMP-8.4 (ranging from 96.7% to 100%), IBMP-8.2 (ranging from 73.3% to 87.5%), and IBMP-8.1 (ranging from 50% to 100%). Considering the merged panel, no statistical differences in sensitivity were observed for IBMP-8.3 and IBMP-8.4. Conversely, IBMP-8.1 showed the lowest sensitivity, mainly for *T*. *cruzi*-positive samples from panel 1 (Sen 50%). False-negative results, produced by this antigen, were due to six dogs infected with the Colombian strain, six with the Y strain, and three with Berenice strain, in which the serum collection occurred two months (nine animals: four infected with Colombian strain, two infected with Y strain, and three infected with Berenice strain), six months (three animals: one and two infected with Colombian and Y strain, respectively), and 15 months (three animals: one infected with Colombian strain and two infected with Y strain) postinfection. IBMP-8.2 also produced a high number of false-negative results, which was due to eight Colombian and one Y strain infected dogs, in which the serum collection occurred two months (three animals infected with Colombian strain), six months (two animals infected with Colombian strain), and 15 months (three animals: two infected with Colombian strain and one with Y strain) postinfection. The performance of IBMP-ELISA evaluated by accuracy showed values of 99% for IBMP-8.4, 98% for IBMP-8.3, and 94% for IBMP-8.2, without significant difference among them. Conversely, IBMP-8.1 was 85.6% accurate, with statistical difference compared with the other antigens (lack of 95% CI values overlapped).

By adopting an inconclusive zone of 1.0 ± 10%, a small number of samples fell inside the grey zone using IBMP-8.2, IBMP-8.3, and IBMP-8.4. However, the RI values of six (2%) *T*. *cruzi*-negative samples fell in the grey zone when assayed with IBMP-8.1. All *T*. *cruzi*-negative samples fell inside the conclusive space when tested using IBMP-8.2 and IBMP-8.4. Two *T*. *cruzi*-negative samples presented inconclusive result when evaluated with IBMP-8.3. With respect to *T*. *cruzi*-positive samples, one sample was inconclusive when assayed with IBMP-8.3 or IBMP-8.4; and two samples tested by IBMP-8.2. Overall analysis showed that 0.33% of the samples assayed using IBMP-8.4, 1% using IBMP-8.2 or IBMP-8.3, and 2% using IBMP-8.1 presented RI values falling within the inconclusive result threshold.

### Analysis of *T*. *cruzi* strain background on antigen efficiency

**Fig 4:**
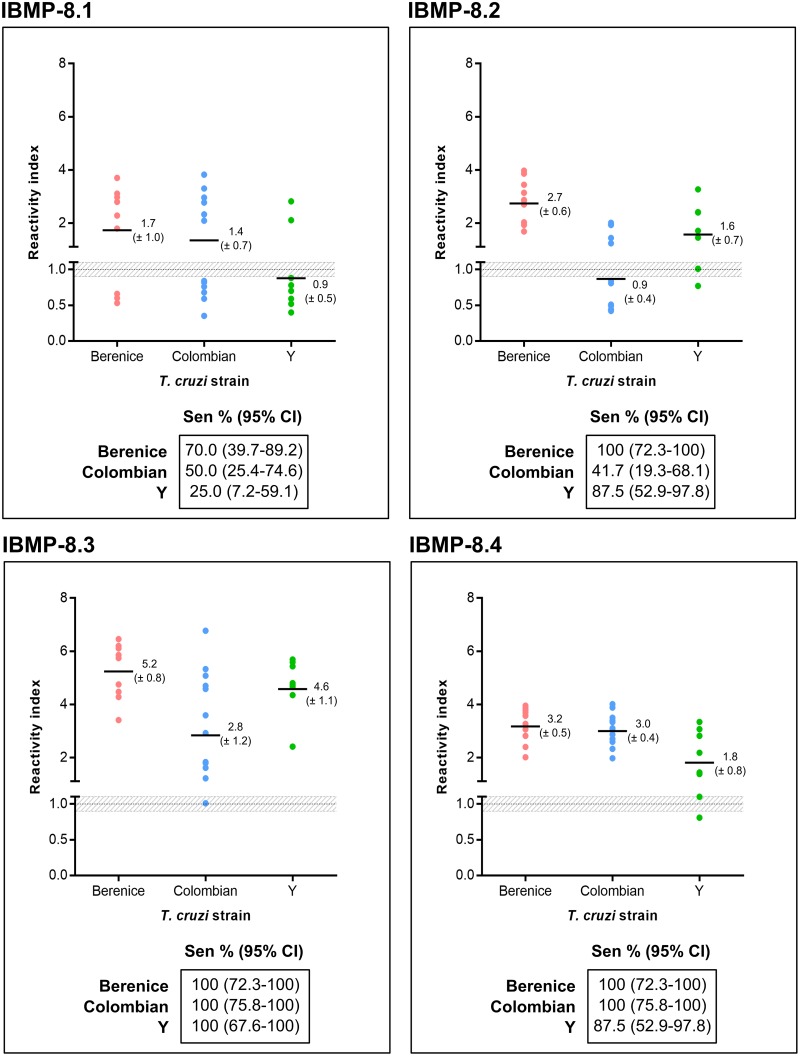
Reactivity index for antigen performance for the different *Trypanosoma cruzi* strains tested. The cut-off is set at the reactivity index value = 1.0 and the shadowed area represents the grey zone (RI = 1.0 ± 0.10). Horizontal lines and numbers for each results group represent the geometric means (± 95% CI). CI (confidence interval); Sen (sensitivity).

In order to evaluate the heterogeneity of recognition of IBMP chimeric antigens by IgG anti-*T*. *cruzi* specific antibodies due to the expected genetic variability of parasite strains, RI and sensitivity values were compared using samples from dogs infected with *T*. *cruzi* Berenice (n = 10), Colombian (n = 12), and Y (n = 8) strains. Consistent with the results described above, the highest RI values were found when *T*. *cruzi*-positive samples were assayed using IBMP-8.3, followed by IBMP-8.4. However, RI values for *T*. *cruzi* Berenice and Y strains were statistically higher for IBMP-8.3 compared to IBMP-8.4 (Fig 4; individual data points are available in the S2 Table). Contrarily, no statistical difference was found when *T*. *cruzi* Colombian strain was assayed with IBMP-8.3 and IBMP-8.4. The lowest RI values were found for IBMP-8.1. Only two out of eight samples were correctly classified as *T*. *cruzi*-positive, producing an RI value of 0.9. Similar RI value was observed when *T*. *cruzi* Colombian strain samples were tested using IBMP-8.2 antigen. As shown in Fig 4, IBMP-8.1 showed the lowest sensitivity value (25%). IBMP-8.2 also showed low sensitivity (41.7%). Higher sensitivity values were observed for IBMP-8.3 (100%) and IBMP-8.4 (87.5–100%).

### Cross-reactivity assessment

**Fig 5:**
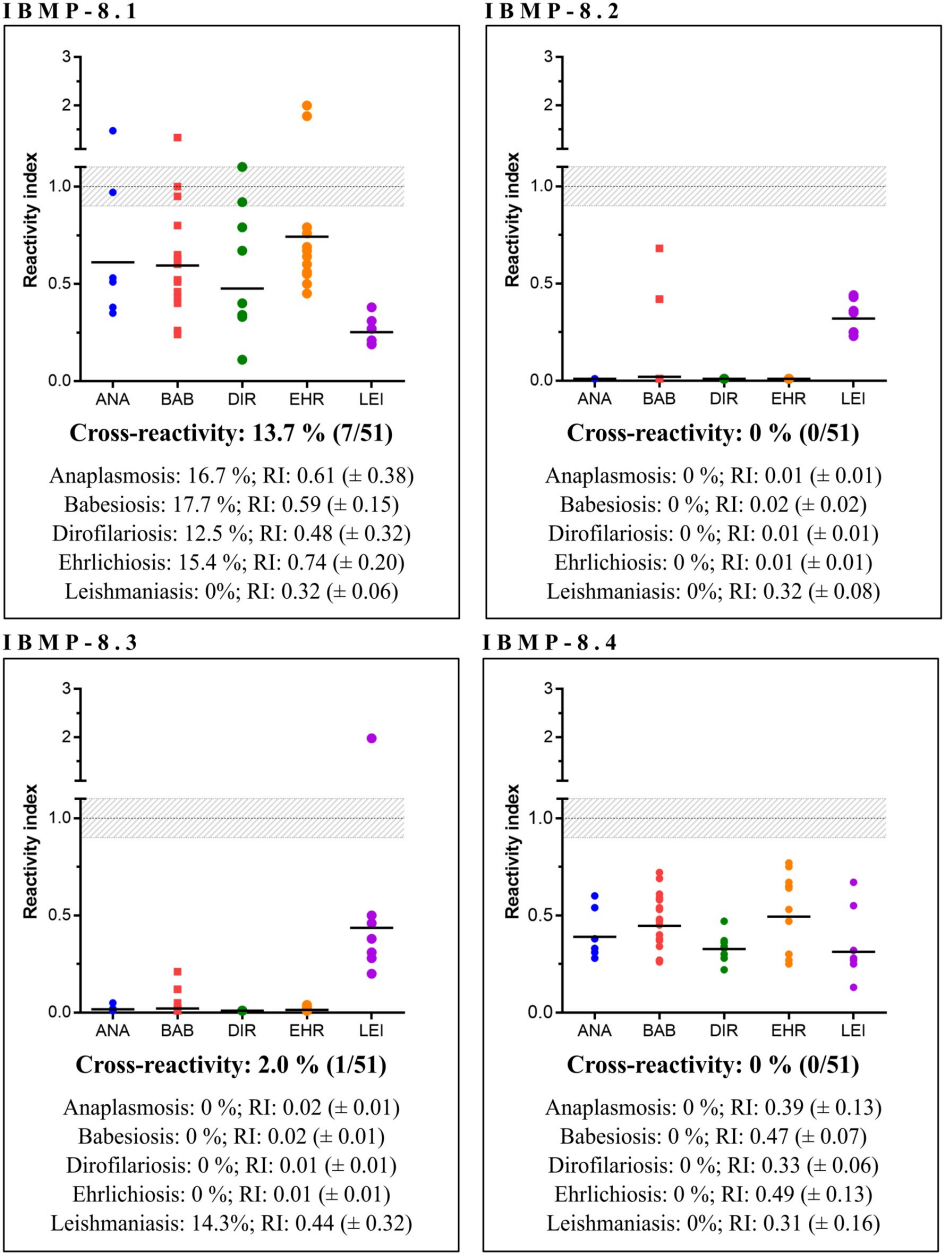
Analysis of IBMP chimeric antigens cross-reactivity with sera from dogs affected by unrelated parasites infection. The cut-off value is reactivity index = 1.0 and the shadowed area represents the grey zone (RI = 1.0 ± 0.10). ANA (anaplasmosis); BAB (babesiosis); DIR (dirofilariosis); EHR (ehrlichiosis); LEI (leishmaniasis).

IBMP-ELISA tests were performed to evaluate the antigenic cross-reactivity against antibodies of unrelated diseases (RI ≥ 1.0) using a panel of 51 serum samples. As shown in Fig 5, no cross-reaction was observed when serum samples were assayed using IBMP-8.2 and IBMP-8.4 (individual RI values are given in the S3 Table). The incidence of cross-reactivity using IBMP-8.3 was negligible; only one *L. infantum*-positive sample produced a false-positive result. Conversely, 13.5% of the samples assayed using IBMP-8.1 presented positive result. At least one sample from each unrelated pathogen cross-reacted with this chimeric antigen, producing a cross-reaction incidence of 16.7% for anaplasmosis, 17.7% for babesiosis, 12.5% for dirofilariosis, and 15.4% for ehrlichiosis. No cross-reaction was observed when *L*. *infantum*-seropositive samples were assayed using IBMP-8.1.

## Discussion

In the present study, we assessed the diagnostic performance of four recombinant chimeric antigens for detection of specific IgG anti-*T*. *cruzi* antibodies in sera from *T*. *cruzi*-positive dogs. The main objectives for surveillance of *T*. *cruzi* infection in domestic animals are to identify mammalian species that can act as amplifiers of parasite populations and to determine the mammalian species that can act as bioindicators (sentinels) of *T*. *cruzi* transmission risk to humans. It is known that, similar to what occurs in *Leishmania* spp. infection, in areas that present a high prevalence of *T*. *cruzi* infection in wild mammalian and triatomine hosts, domestic and peridomestic (synanthropic) mammalian species are exposed to infection, a scenario that favors establishment of human CD in that same area [7]. The antigens assayed in this work exhibited a high diagnostic efficiency. Indeed, the AUC values were higher than 90% for all antigens, indicating an optimal discriminative power between *T*. *cruzi*-positive and negative canine sera. IBMP-8.3 and IBMP-8.4 are of particular interest since they presented AUC values of 99.7% and 100%, respectively. These data are similar to previous results obtained by our group when these *T*. *cruzi* antigens were assessed to diagnose CD in humans [18–20]. Currently, human chronic CD diagnosis is troublesome due to the lack of a gold-standard serological test and the only gold standard tests based on *T*. *cruzi* DNA detection are solely applicable in the brief initial stage of the infection (acute phase). However, there is currently a significant difference in performance among the commercial tests due to the high genetic variability of the parasite and the employed antigenic matrices used to capture specific antibodies [18,33]. Hence, the World Health Organization recommends the concomitant use of two antigenically distinct commercial tests to diagnose *T*. *cruzi* infection in humans [34]. The diagnosis of the *T*. *cruzi* infection in dogs is even more difficult, owing to the absence of validated tests. Most studies usually employ either fractionated *T*. *cruzi* lysates or whole-cell epimastigote homogenates as antigenic matrices [10,13–15], which can lead to difficulties in standardizing, low specificity, and cross-reactivity with antibodies against *Leishmania* spp., amongst other trypanosomatids [16,17]. So, a strategy to address these limitations can be proposed by adopting synthetic recombinant chimeric antigens, composed of conserved amino acid sequences of several antigenic *T*. *cruzi* proteins [21,23,35,36]. To the best of our knowledge, this is the first study using recombinant chimeric proteins to diagnose *T*. *cruzi* infection in experimentally infected animals with known strains.

The diagnostic sensitivity was higher for IBMP-8.3 and IBMP-8.4 compared to the other chimeric proteins. Indeed, IBMP-8.3 correctly diagnosed all *T*. *cruzi* positive samples, regardless of the panel or strain, whereas IBMP-8.4 misidentified as negative only one positive sample from a dog infected with the Y strain. Conversely, IBMP-8.1 and IBMP-8.2 presented lower sensitivity values, probably due to their amino acid composition and the short period between inoculation and sampling (acute phase). We observed that the majority of false-negative results produced by IBMP-8.1 and IBMP-8.2 was associated with samples from panel 1. This panel was composed of dogs submitted to well-established infection protocols in which known strains were used. The sera sampling occurred two, six or 15 months postinfection. We observed that almost 50% of the false-negative results occurred in animals whose samples were collected two months postinfection. Thus, the quantity of specific IgG anti-*T*. *cruzi* antibodies did not appear sufficient to be detected by the IBMP-8.1 and IBMP-8.2 immunoassays performed here. Furthermore, the limited repertoire of antigenic epitopes in IBMP-8.1 and IBMP-8.2 compared to IBMP-8.3 and IBMP-8.4 could not be wide enough to identify the specific antibodies.

Considering the cross-reactivity assessment with sera from dogs carrying unrelated pathogens, the small number of samples that cross-reacted was statistically insignificant for the assays with IBMP-8.2, IBMP-8.3, and IBMP-8.4. IBMP-8.3 cross-reacted with one *Leishmania infantum*-positive serum, which was negative for other chimeric antigens. Conversely, IBMP-8.1 recognized at least one serum for anaplasmosis, babesiosis, dirofilariosis and ehrlichiosis as *T*. *cruzi*-positive samples, producing an overall cross-reaction rate of 13.7%. This was not expected owing to the low similarity of IBMP antigen sequences of *T*. *cruzi* to those deposited in the NCBI’s Genbank for other species. Furthermore, cross-reacting samples also presented a low RI value. Inconclusive results using this panel were statistically insignificant, except to IBMP-8.1. These findings are similar to previous results obtained when our group assessed the cross-reactivity in human samples for pathogens of medical interest, such as dengue, B and C hepatitis, HIV, HTLV, visceral and cutaneous leishmaniasis, leptospirosis, rubella, measles, schistosomiasis, and syphilis using both ELISA and liquid microarray [19,20,26]. The low number of cross-reaction suggests that IBMP antigens, specially IBMP-8.3, can be safely used to diagnose canine *T*. *cruzi* infection in co-endemicity areas with other infectious parasites.

The main limitation of the study was the lack of a validated standard test to pre-classify the sera to be used to evaluate the efficiency of the antigens. To overcome this limitation we employed two in-house ELISAs as reference tests. So, the diagnostic performance of the present IBMP chimeric antigens could be biased due to shortcomings in the accuracy of the reference test. Another limitation was the number of samples with unrelated pathogens. Despite these restrictions, we conclude that IBMP-8.3 and IBMP-8.4 can be used for anti-*T*. *cruzi* IgG antibodies detection in dogs. These antigens could be potentially employed in a test to evaluate parasite’s transmission cycle of *T*. *cruzi* in endemic settings and for veterinary purposes.

## Supporting information

S1 Table STARD checklist. Standards for the Reporting of Diagnostic Accuracy Studies (STARD) checklist for reporting of studies of diagnostic accuracy.
(PDF)

S2 Table Reactivity index for diagnostic performance assessment. (PDF)

S3 Table Reactivity index for cross-reactivity assessment. (PDF)
