# Supplementary material for: Performance of recombinant chimeric proteins in the serological diagnosis of Trypanosoma cruzi infection in dogs
Source: PLoS Negl Trop Dis. 2019 Jun 26;13(6):e0007545. doi: 10.1371/journal.pntd.0007545 (PMC6615644; doi:10.1371/journal.pntd.0007545)
Supplement: S2 Table — (PDF) [file pntd.0007545.s002.pdf]

Sample	Panel	Strain	Reactivity Index			
			IBMP-8.1	IBMP-8.2	IBMP-8.3	IBMP-8.4
T. cruzi-positive_1	1	Colombian	0.68	0.51	1.79	1.97
T. cruzi-positive_2	1	Colombian	0.84	1.44	2.92	2.70
T. cruzi-positive_3	1	Colombian	0.76	0.81	1.22	2.59
T. cruzi-positive_4	1	Colombian	0.35	0.44	1.01	2.33
T. cruzi-positive_5	1	Colombian	2.96	0.84	4.70	3.33
T. cruzi-positive_6	1	Colombian	2.77	1.93	5.08	3.50
T. cruzi-positive_7	1	Colombian	3.82	2.01	6.77	3.88
T. cruzi-positive_8	1	Colombian	0.82	0.44	1.83	2.87
T. cruzi-positive_9	1	Colombian	2.33	0.42	5.33	3.39
T. cruzi-positive_10	1	Colombian	2.09	1.24	4.58	3.10
T. cruzi-positive_11	1	Colombian	3.30	1.94	3.59	4.01
T. cruzi-positive_12	1	Colombian	0.59	0.49	1.61	3.03
T. cruzi-positive_13	1	Y	0.78	2.40	4.73	2.82
T. cruzi-positive_14	1	Y	0.52	1.45	2.41	1.44
T. cruzi-positive_15	1	Y	0.59	1.01	4.66	0.81
T. cruzi-positive_16	1	Y	0.88	1.71	5.59	2.18
T. cruzi-positive_17	1	Y	2.82	3.27	4.80	3.34
T. cruzi-positive_18	1	Y	2.11	2.41	4.35	3.07
T. cruzi-positive_19	1	Y	0.40	0.77	5.43	1.10
T. cruzi-positive_20	1	Y	0.70	1.01	5.69	1.38
T. cruzi-positive_21	1	Berenice	0.53	3.44	5.74	3.06
T. cruzi-positive_22	1	Berenice	0.66	1.93	4.47	2.40
T. cruzi-positive_23	1	Berenice	0.60	1.68	3.41	2.01
T. cruzi-positive_24	1	Berenice	2.97	3.86	6.11	3.77
T. cruzi-positive_25	1	Berenice	2.28	3.97	4.75	3.27
T. cruzi-positive_26	1	Berenice	3.70	3.14	6.46	3.68
T. cruzi-positive_27	1	Berenice	3.11	2.87	6.12	2.82
T. cruzi-positive_28	1	Berenice	1.79	2.70	5.86	3.57
T. cruzi-positive_29	1	Berenice	2.95	2.85	6.20	3.95
T. cruzi-positive_30	1	Berenice	2.80	2.03	4.28	3.86
T. cruzi-negative_1	1	Not applicable	0.11	0.20	0.33	0.19
T. cruzi-negative_2	1	Not applicable	0.27	0.26	0.27	0.27
T. cruzi-negative_3	1	Not applicable	0.17	0.28	0.19	0.30
T. cruzi-negative_4	1	Not applicable	0.10	0.16	0.29	0.16
T. cruzi-negative_5	1	Not applicable	0.21	0.22	0.26	0.20
T. cruzi-negative_6	1	Not applicable	0.25	0.37	0.02	0.02
T. cruzi-negative_7	1	Not applicable	0.31	0.33	0.23	0.18
T. cruzi-negative_8	1	Not applicable	0.21	0.19	0.23	0.28
T. cruzi-negative_9	1	Not applicable	0.26	0.26	0.52	0.29
T. cruzi-negative_10	1	Not applicable	0.29	0.22	0.33	0.35
T. cruzi-negative_11	1	Not applicable	0.24	0.26	0.47	0.50
T. cruzi-negative_12	1	Not applicable	0.16	0.17	0.32	0.29

Sample	Panel	Strain	Reactivity Index			
			IBMP-8.1	IBMP-8.2	IBMP-8.3	IBMP-8.4
T. cruzi-negative_13	1	Not applicable	0.24	0.23	0.35	0.21
T. cruzi-negative_14	1	Not applicable	0.34	0.48	0.63	0.31
T. cruzi-negative_15	1	Not applicable	0.38	0.37	0.54	0.32
T. cruzi-negative_16	1	Not applicable	0.31	0.25	0.42	0.37
T. cruzi-negative_17	1	Not applicable	0.20	0.22	0.35	0.28
T. cruzi-negative_18	1	Not applicable	0.51	0.47	0.59	0.48
T. cruzi-negative_19	1	Not applicable	0.24	0.25	0.89	0.49
T. cruzi-negative_20	1	Not applicable	0.31	0.35	0.82	0.55
T. cruzi-negative_21	1	Not applicable	0.21	0.39	0.45	0.23
T. cruzi-negative_22	1	Not applicable	0.53	0.54	1.21	0.59
T. cruzi-negative_23	1	Not applicable	0.45	0.45	0.40	0.56
T. cruzi-negative_24	1	Not applicable	0.21	0.20	0.44	0.26
T. cruzi-negative_25	1	Not applicable	0.22	0.18	0.31	0.57
T. cruzi-negative_26	1	Not applicable	0.22	0.23	0.20	0.34
T. cruzi-negative_27	1	Not applicable	0.18	0.29	0.26	0.24
T. cruzi-negative_28	1	Not applicable	0.22	0.36	0.07	0.19
T. cruzi-negative_29	1	Not applicable	0.26	0.34	0.40	0.25
T. cruzi-negative_30	1	Not applicable	0.19	0.25	0.31	0.27
T. cruzi-positive_31	2	Unknown	1.29	1.97	2.24	2.27
T. cruzi-positive_32	2	Unknown	2.04	1.95	1.74	1.64
T. cruzi-positive_33	2	Unknown	2.05	0.59	2.80	2.19
T. cruzi-positive_34	2	Unknown	1.63	1.82	1.48	2.79
T. cruzi-positive_35	2	Unknown	1.34	3.99	3.16	1.73
T. cruzi-positive_36	2	Unknown	2.87	1.99	1.27	1.97
T. cruzi-positive_37	2	Unknown	1.68	1.58	2.60	1.70
T. cruzi-positive_38	2	Unknown	1.36	1.86	1.87	1.51
T. cruzi-positive_39	2	Unknown	1.26	1.11	3.37	4.30
T. cruzi-positive_40	2	Unknown	1.79	2.76	1.63	4.57
T. cruzi-positive_41	2	Unknown	1.33	1.38	1.60	4.55
T. cruzi-positive_42	2	Unknown	2.08	2.06	3.92	4.36
T. cruzi-positive_43	2	Unknown	2.20	1.91	1.50	3.55
T. cruzi-positive_44	2	Unknown	1.62	1.92	1.64	4.13
T. cruzi-positive_45	2	Unknown	2.38	1.83	1.53	1.90
T. cruzi-positive_46	2	Unknown	1.60	0.96	3.78	4.99
T. cruzi-negative_31	2	Not applicable	0.11	0.34	0.35	0.25
T. cruzi-negative_32	2	Not applicable	0.49	0.68	0.54	0.50
T. cruzi-negative_33	2	Not applicable	0.21	0.48	0.39	0.40
T. cruzi-negative_34	2	Not applicable	0.69	0.11	0.97	0.31
T. cruzi-negative_35	2	Not applicable	0.71	0.46	0.51	0.46
T. cruzi-negative_36	2	Not applicable	0.67	0.78	0.78	0.28
T. cruzi-negative_37	2	Not applicable	0.26	0.48	0.35	0.50

Sample	Panel	Strain	Reactivity Index			
			IBMP-8.1	IBMP-8.2	IBMP-8.3	IBMP-8.4
T. cruzi-negative_38	2	Not applicable	0.37	0.60	0.56	0.18
T. cruzi-negative_39	2	Not applicable	0.29	0.50	0.41	0.69
T. cruzi-negative_40	2	Not applicable	0.48	0.67	0.46	0.57
T. cruzi-negative_41	2	Not applicable	0.43	0.58	0.46	0.60
T. cruzi-negative_42	2	Not applicable	0.20	0.33	0.28	0.32
T. cruzi-negative_43	2	Not applicable	0.20	0.34	0.25	0.37
T. cruzi-negative_44	2	Not applicable	0.54	0.59	1.04	0.24
T. cruzi-negative_45	2	Not applicable	0.35	0.46	0.36	0.50
T. cruzi-negative_46	2	Not applicable	0.23	0.39	0.37	0.43
T. cruzi-negative_47	2	Not applicable	0.33	0.54	0.45	0.63
T. cruzi-negative_48	2	Not applicable	0.17	0.29	0.29	0.38
T. cruzi-negative_49	2	Not applicable	0.33	0.45	0.32	0.43
T. cruzi-negative_50	2	Not applicable	1.67	0.39	0.32	0.41
T. cruzi-negative_51	2	Not applicable	0.08	0.53	0.09	0.16
T. cruzi-negative_52	2	Not applicable	0.30	0.29	0.34	0.44
T. cruzi-negative_53	2	Not applicable	0.41	0.09	0.44	0.53
T. cruzi-negative_54	2	Not applicable	0.19	0.62	0.18	0.28
T. cruzi-negative_55	2	Not applicable	0.66	0.58	0.45	0.45
T. cruzi-negative_56	2	Not applicable	1.33	0.09	1.97	0.68
T. cruzi-negative_57	2	Not applicable	0.24	0.45	0.39	0.45
T. cruzi-negative_58	2	Not applicable	0.24	0.14	0.34	0.41
T. cruzi-negative_59	2	Not applicable	0.31	0.41	0.29	0.35
T. cruzi-negative_60	2	Not applicable	0.96	0.04	0.29	0.38
T. cruzi-negative_61	2	Not applicable	0.22	0.61	0.32	0.38
T. cruzi-negative_62	2	Not applicable	0.31	0.58	0.38	0.59
T. cruzi-negative_63	2	Not applicable	0.04	0.28	0.03	0.05
T. cruzi-negative_64	2	Not applicable	0.30	0.48	0.30	0.47
T. cruzi-negative_65	2	Not applicable	0.82	0.47	0.29	0.57
T. cruzi-negative_66	2	Not applicable	0.35	0.51	0.43	0.62
T. cruzi-negative_67	2	Not applicable	0.32	0.43	0.41	0.37
T. cruzi-negative_68	2	Not applicable	0.09	0.18	0.26	0.20
T. cruzi-negative_69	2	Not applicable	0.41	0.51	0.60	0.52
T. cruzi-negative_70	2	Not applicable	0.27	0.34	0.10	0.40
T. cruzi-negative_71	2	Not applicable	0.35	0.44	0.14	0.57
T. cruzi-negative_72	2	Not applicable	0.15	0.45	0.54	0.75
T. cruzi-negative_73	2	Not applicable	0.15	0.01	0.07	0.11
T. cruzi-negative_74	2	Not applicable	0.32	0.45	0.36	0.47
T. cruzi-negative_75	2	Not applicable	0.50	0.14	0.46	0.43
T. cruzi-negative_76	2	Not applicable	0.40	0.41	0.47	0.53
T. cruzi-negative_77	2	Not applicable	0.76	0.08	0.72	0.51

Sample	Panel	Strain	Reactivity Index			
			IBMP-8.1	IBMP-8.2	IBMP-8.3	IBMP-8.4
T. cruzi-negative_78	2	Not applicable	0.08	0.11	0.04	0.09
T. cruzi-negative_79	2	Not applicable	0.66	0.68	0.25	0.20
T. cruzi-negative_80	2	Not applicable	0.40	0.35	0.15	0.08
T. cruzi-negative_81	2	Not applicable	0.44	0.37	0.11	0.14
T. cruzi-negative_82	2	Not applicable	0.88	0.56	0.32	0.23
T. cruzi-negative_83	2	Not applicable	0.34	0.35	0.16	0.11
T. cruzi-negative_84	2	Not applicable	0.21	0.27	0.11	0.09
T. cruzi-negative_85	2	Not applicable	0.29	0.25	0.10	0.11
T. cruzi-negative_86	2	Not applicable	0.36	0.58	0.38	0.24
T. cruzi-negative_87	2	Not applicable	0.30	0.35	0.16	0.12
T. cruzi-negative_88	2	Not applicable	0.64	0.66	0.21	0.19
T. cruzi-negative_89	2	Not applicable	0.32	0.40	0.14	0.01
T. cruzi-negative_90	2	Not applicable	1.02	0.70	0.25	0.20
T. cruzi-negative_91	2	Not applicable	0.54	0.45	0.26	0.40
T. cruzi-negative_92	2	Not applicable	1.04	0.57	0.20	0.16
T. cruzi-negative_93	2	Not applicable	0.45	0.51	0.19	0.15
T. cruzi-negative_94	2	Not applicable	1.74	0.75	0.46	0.32
T. cruzi-negative_95	2	Not applicable	0.36	0.36	0.14	0.13
T. cruzi-negative_96	2	Not applicable	0.99	0.49	0.31	0.16
T. cruzi-negative_97	2	Not applicable	1.20	0.73	0.49	0.46
T. cruzi-negative_98	2	Not applicable	0.46	0.43	0.18	0.17
T. cruzi-negative_99	2	Not applicable	0.63	0.51	0.17	0.18
T. cruzi-negative_100	2	Not applicable	0.94	0.48	0.30	0.25
T. cruzi-negative_101	2	Not applicable	0.39	0.01	0.13	0.10
T. cruzi-negative_102	2	Not applicable	1.60	0.22	0.77	0.57
T. cruzi-negative_103	2	Not applicable	0.72	0.54	0.18	0.13
T. cruzi-negative_104	2	Not applicable	0.36	0.31	0.13	0.13
T. cruzi-negative_105	2	Not applicable	0.21	0.26	0.08	0.12
T. cruzi-negative_106	2	Not applicable	0.42	0.51	0.18	0.22
T. cruzi-negative_107	2	Not applicable	0.68	0.44	0.16	0.17
T. cruzi-negative_108	2	Not applicable	1.52	0.22	0.68	0.61
T. cruzi-negative_109	2	Not applicable	1.32	0.83	0.77	0.47
T. cruzi-negative_110	2	Not applicable	0.26	0.28	0.10	0.11
T. cruzi-negative_111	2	Not applicable	0.33	0.44	0.14	0.17
T. cruzi-negative_112	2	Not applicable	0.23	0.29	0.08	0.01
T. cruzi-negative_113	2	Not applicable	0.41	0.42	0.12	0.15
T. cruzi-negative_114	2	Not applicable	0.63	0.66	0.22	0.14
T. cruzi-negative_115	2	Not applicable	0.33	0.25	0.08	0.09
T. cruzi-negative_116	2	Not applicable	0.72	0.54	0.20	0.18
T. cruzi-negative_117	2	Not applicable	0.40	0.29	0.08	0.14

Sample	Panel	Strain	Reactivity Index			
			IBMP-8.1	IBMP-8.2	IBMP-8.3	IBMP-8.4
T. cruzi-negative_118	2	Not applicable	0.72	0.42	0.12	0.21
T. cruzi-negative_119	2	Not applicable	0.73	0.34	0.10	0.22
T. cruzi-negative_120	2	Not applicable	0.45	0.66	0.22	0.17
T. cruzi-negative_121	2	Not applicable	0.64	0.54	0.20	0.33
T. cruzi-negative_122	2	Not applicable	0.62	0.50	0.16	0.22
T. cruzi-negative_123	2	Not applicable	0.43	0.69	0.17	0.14
T. cruzi-negative_124	2	Not applicable	0.41	0.76	0.21	0.15
T. cruzi-negative_125	2	Not applicable	0.39	0.60	0.21	0.13
T. cruzi-negative_126	2	Not applicable	1.68	0.45	0.12	0.56
T. cruzi-negative_127	2	Not applicable	0.58	0.70	0.25	0.14
T. cruzi-negative_128	2	Not applicable	0.87	0.39	0.10	0.17
T. cruzi-negative_129	2	Not applicable	0.74	0.69	0.17	0.11
T. cruzi-negative_130	2	Not applicable	0.62	0.76	0.21	0.20
T. cruzi-negative_131	2	Not applicable	0.55	0.60	0.21	0.20
T. cruzi-negative_132	2	Not applicable	0.81	0.68	0.21	0.25
T. cruzi-negative_133	2	Not applicable	1.09	0.45	0.12	0.55
T. cruzi-negative_134	2	Not applicable	0.65	0.79	0.20	0.26
T. cruzi-negative_135	2	Not applicable	0.30	0.32	0.09	0.10
T. cruzi-negative_136	2	Not applicable	0.62	0.61	0.15	0.18
T. cruzi-negative_137	2	Not applicable	0.47	0.35	0.37	0.38
T. cruzi-negative_138	2	Not applicable	1.20	0.20	0.46	0.41
T. cruzi-negative_139	2	Not applicable	0.69	0.47	0.24	0.25
T. cruzi-negative_140	2	Not applicable	0.31	0.58	0.17	0.24
T. cruzi-negative_141	2	Not applicable	0.33	0.47	0.20	0.12
T. cruzi-negative_142	2	Not applicable	1.51	0.61	0.18	0.25
T. cruzi-negative_143	2	Not applicable	0.24	0.33	0.36	0.12
T. cruzi-negative_144	2	Not applicable	0.53	0.53	0.18	0.02
T. cruzi-negative_145	2	Not applicable	0.48	0.52	0.57	0.64
T. cruzi-negative_146	2	Not applicable	0.31	0.34	0.39	0.30
T. cruzi-negative_147	2	Not applicable	0.21	0.30	0.34	0.34
T. cruzi-negative_148	2	Not applicable	0.61	0.63	0.40	0.60
T. cruzi-negative_149	2	Not applicable	0.80	0.61	1.40	0.44
