# Supplementary material for: Performance of recombinant chimeric proteins in the serological diagnosis of Trypanosoma cruzi infection in dogs
Source: PLoS Negl Trop Dis. 2019 Jun 26;13(6):e0007545. doi: 10.1371/journal.pntd.0007545 (PMC6615644; doi:10.1371/journal.pntd.0007545)
Supplement: S3 Table — (PDF) [file pntd.0007545.s003.pdf]

Sample	Panel	Reactivity Index			
		IBMP-8.1	IBMP-8.2	IBMP-8.3	IBMP-8.4
Anaplasmosis_1	3	0.51	0.01	0.01	0.54
Anaplasmosis_2	3	0.35	0.01	0.01	0.33
Anaplasmosis_3	3	0.53	0.01	0.02	0.31
Anaplasmosis_4	3	1.48	0.01	0.05	0.60
Anaplasmosis_5	3	0.97	0.01	0.02	0.38
Anaplasmosis_6	3	0.38	0.01	0.01	0.28
Babesiosis_1	3	0.65	0.42	0.12	0.69
Babesiosis_2	3	0.52	0.68	0.21	0.47
Babesiosis_3	3	0.52	0.42	0.12	0.53
Babesiosis_4	3	1.34	0.01	0.01	0.34
Babesiosis_5	3	1.34	0.01	0.01	0.48
Babesiosis_6	3	0.46	0.01	0.01	0.45
Babesiosis_7	3	1.00	0.01	0.05	0.37
Babesiosis_8	3	0.63	0.01	0.01	0.59
Babesiosis_9	3	0.24	0.01	0.02	0.27
Babesiosis_10	3	0.95	0.01	0.02	0.72
Babesiosis_11	3	0.40	0.01	0.01	0.40
Babesiosis_12	3	0.43	0.01	0.03	0.38
Babesiosis_13	3	0.60	0.01	0.01	0.61
Babesiosis_14	3	0.80	0.01	0.01	0.27
Babesiosis_15	3	0.63	0.01	0.01	0.58
Babesiosis_16	3	0.51	0.01	0.02	0.54
Babesiosis_17	3	0.26	0.01	0.01	0.26
Dirofilariasis_1	3	0.79	0.01	0.01	0.22
Dirofilariasis_2	3	0.67	0.01	0.01	0.36
Dirofilariasis_3	3	0.92	0.01	0.01	0.28
Dirofilariasis_4	3	0.34	0.01	0.01	0.34
Dirofilariasis_5	3	0.11	0.01	0.01	0.30
Dirofilariasis_6	3	1.10	0.01	0.01	0.47
Dirofilariasis_7	3	0.33	0.01	0.01	0.33
Dirofilariasis_8	3	0.40	0.01	0.01	0.37
Ehrlichiosis_1	3	0.45	0.01	0.01	0.25
Ehrlichiosis_2	3	0.69	0.01	0.01	0.27
Ehrlichiosis_3	3	0.64	0.01	0.01	0.65
Ehrlichiosis_4	3	0.55	0.01	0.01	0.47
Ehrlichiosis_5	3	0.60	0.01	0.01	0.65
Ehrlichiosis_6	3	0.50	0.01	0.01	0.64
Ehrlichiosis_7	3	0.76	0.01	0.02	0.77
Ehrlichiosis_8	3	2.00	0.01	0.02	0.30
Ehrlichiosis_9	3	0.79	0.01	0.02	0.30
Ehrlichiosis_10	3	1.78	0.01	0.04	0.75
Ehrlichiosis_11	3	0.79	0.01	0.01	0.65
Ehrlichiosis_12	3	0.56	0.01	0.01	0.53
Ehrlichiosis_13	3	0.67	0.01	0.03	0.67
Leishmaniasis_1	3	0.38	0.43	1.98	0.67
Leishmaniasis_2	3	0.19	0.25	0.20	0.13
Leishmaniasis_3	3	0.21	0.25	0.31	0.25
Leishmaniasis_4	3	0.27	0.44	0.46	0.32
Leishmaniasis_5	3	0.19	0.23	0.38	0.28
Leishmaniasis_6	3	0.31	0.35	0.28	0.55
Leishmaniasis_7	3	0.27	0.36	0.50	0.27
